# Psychological Contract Violation or Basic Need Frustration? Psychological Mechanisms Behind the Effects of Workplace Bullying

**DOI:** 10.3389/fpsyg.2021.627968

**Published:** 2021-04-09

**Authors:** Philipp E. Sischka, André Melzer, Alexander F. Schmidt, Georges Steffgen

**Affiliations:** ^1^Department of Behavioural and Cognitive Sciences, Institute for Health and Behaviour, University of Luxembourg, Esch-sur-Alzette, Luxembourg; ^2^Department of Psychology, Social and Legal Psychology, University of Mainz, Mainz, Germany

**Keywords:** workplace bullying, job satisfaction, well-being, turnover intentions, psychological contract violation, basic need frustration, self-determination theory

## Abstract

Workplace bullying is a phenomenon that can have serious detrimental effects on health, work-related attitudes, and the behavior of the target. Particularly, workplace bullying exposure has been linked to lower level of general well-being, job satisfaction, vigor, and performance and higher level of burnout, workplace deviance, and turnover intentions. However, the psychological mechanisms behind these relations are still not well-understood. Drawing on psychological contract and self-determination theory (SDT), we hypothesized that perceptions of contract violation and the frustration of basic needs mediate the relationship between workplace bullying exposure and well-being, attitudinal, and behavioral outcomes. Self-reported data were collected among employees with different working backgrounds (*N* = 1,257) *via* Amazon's Mechanical Turk in an online survey. Results showed that feelings of contract violation and frustration of basic needs accounted for unique variation in well-being, work satisfaction, burnout, vigor, and turnover intentions, pointing to individual contributions of both psychological mechanisms. However, when controlled for frustration of basic needs, feelings of psychological contract violation were no longer a mediator between workplace bullying exposure and work performance. Helping employees to deal effectively with workplace bullying exposure might buffer its negative effects and reduce their experienced frustration of basic needs, preserving their well-being, vigor, and work performance and, eventually, prevent burnout. The present study is the first to concurrently elucidate the proposed psychological mechanisms and unique contributions of psychological contract violation and frustration of basic needs in the context of workplace bullying.

## Introduction

An impressive number of studies on workplace bullying have shown its detrimental effects on victim's health, work-related attitudes, and behavior (e.g., Nielsen and Einarsen, 2012; Steffgen et al., 2019). Workplace bullying describes a situation where an employee persistently and over a period of time perceives himself/herself to be on the receiving end of negative treatments from people at work (i.e., colleagues, supervisor, subordinates, customer, clients) while finding it difficult to defend himself/herself against these negative treatments (Einarsen and Skogstad, 1996)^1^. Prolonged exposure to bullying experiences at the workplace has been shown to decrease general mental health and job satisfaction and to increase burnout (e.g., Dehue et al., 2012; Nielsen and Einarsen, 2012; Raja et al., 2018). Furthermore, workplace bullying exposure has been linked with a decrease of vigor (Rodríguez-Muñoz et al., 2015), work performance (Bowling and Beehr, 2006), workplace deviance (Bowling and Beehr, 2006), and turnover intentions (Nielsen and Einarsen, 2012).

Despite these well-documented detrimental effects, researchers have only recently begun to investigate the psychological mechanisms underlying the relationships between workplace bullying exposure and its various negative outcomes (e.g., Salin and Notelaers, 2017). Studies on the possible mechanisms (e.g., mediators and moderators) between being target of workplace bullying and various outcomes (e.g., well-being, job satisfaction, vigor, subjective work performance, burnout, workplace deviance, and turnover intentions) are still sparse, and there have been repeated calls for studies that research the *when* and *how* of bullying-outcome-relationships (Nielsen and Einarsen, 2018; Rai and Agarwal, 2018). Specifically, two psychological theories have gained attention to explain the link between workplace bullying exposure and its negative consequences: psychological contract theory (e.g., Salin and Notelaers, 2017) and the self-determination theory (SDT; e.g., Trépanier et al., 2016). As workplace bullying concerns perceptions of behaviors that appear in a relationship in the workplace context, one might argue that social exchange theory-based concepts, such as psychological contract violation, can explain the link between bullying exposure and certain outcomes (Parzefall and Salin, 2010). On the other hand, SDT proposes that need-thwarting environments consisting of controlling, critical, or rejecting social contexts contribute to individuals malfunctioning and ill-being (Vansteenkiste and Ryan, 2013). Being on the receiving end of these negative acts that aim to personally harm the target might represent a need-thwarting situation (e.g., Trépanier et al., 2016).

Although these possibly mediating mechanisms between workplace bullying exposure and different outcomes have been investigated separately, to date they have not been jointly tested. Therefore, it cannot be ruled out that some detected mediation effects may be due to confounders of psychological contract violation and frustration of basic psychological needs. It may well be that there is no incremental explanatory power of one theory above and beyond the other. This bears the risk to theory inflation and, ultimately, misinform possible intervention strategies. To this end, we will argue that the two constructs are incremental mediators that link workplace bullying exposure to various negative outcomes.

The present study makes several contributions. First, we present theoretical considerations and empirical findings on *why* psychological contract violation and frustration of basic psychological needs may act as (independent) mediators between workplace bullying exposure and different outcomes (i.e., general well-being, job satisfaction, vigor, subjective job performance, burnout, workplace deviance, and turnover intentions). Second, we subsequently investigate which of the proposed mechanisms best explain the associations between workplace bullying exposure and the different outcome variables. To this end, the two hypothesized mechanisms were simultaneously tested to elucidate their independent contributions to the outcomes once controlled for the other mediator. Third, in contrast to previous studies, we study need frustration in contrast to need satisfaction. This is a critical issue as low need satisfaction may not relate as robustly to malfunctioning and ill-being as frustrated needs (Vansteenkiste and Ryan, 2013). As workplace bullying exposure is associated with personal harm (Tuckey et al., 2015), investigating need frustration is a more direct test of the hypothesis regarding basic psychological needs as potential mediators in the context of workplace bullying exposure. Finally, we also include a range of different (well-being, attitudinal, behavioral) outcome variables, some (i.e., general well-being, job performance, workplace deviance) that so far have not been researched as an outcome in these mediation models (i.e., workplace bullying exposure as predictor, psychological contract violation, and frustration of basic psychological needs as mediators). The following sections present the theoretical framework for the development of our workplace bullying model and hypotheses.

### Workplace Bullying and the Psychological Contract

The idea of psychological contract is based on implicit beliefs about the promises and commitments made in the exchange relationship (Rousseau, 1995). A psychological contract is shaped through pre-employment schemas, the recruitment process, and post-hire socialization (Rousseau, 2001). In contrast to formalized contracts, psychological contracts are thus only informal and often implicit, and the perception and interpretation of the other's attitude and behavior play a central role (Salin and Notelaers, 2017). Psychological contract breach refers to the perception of failure to fulfill these promises. A meta-analysis confirmed the negative consequences of a perceived psychological contract breach on work attitudes and behavior, including job satisfaction, in-role performance, and turnover intentions (Zhao et al., 2007). According to Robinson and Morrison (2000), psychological contract breach should even lead to more negative effects, when these perceptions are related to emotional reactions of anger and betrayal (i.e., feeling of psychological contract violation). In fact, a number of studies have shown that the feeling of psychological contract violation (i.e., frustration, anger, bitterness, and feelings of betrayal directed at the organization) is an important mediator between contract breach and various negative outcomes (Zhao et al., 2007; Robbins et al., 2012). These affective reactions can be framed as antecedents of work-related health, attitudes, and behavior (Weiss and Cropanzano, 1996). As job satisfaction is a function of the discrepancy between what an employee expects from his/her job and what he/she perceives as offering, feelings of psychological contract violation decrease job satisfaction (Zhao et al., 2007). Furthermore, if the job is valued less as a result of feelings of psychological contract violation, turnover intentions increase as it can be regarded as an indicator of the employee's psychological attachment to the organization (Zhao et al., 2007). Because feelings of psychological contract violation consist of negative emotions, they also have an impact on emotional well-being (Cassar and Buttigieg, 2015). Additionally, employees with negative emotions due to psychological contract violation are less likely to feel dedicated or energetic to help the organization to reach its goals (Rai and Agarwal, 2017). According to the norm of reciprocity, employees reduce their efforts as a reaction of a perceived contract violation, resulting in lower job performance (Bal et al., 2010). Finally, feelings of violation can even initiate revenge seeking in order to “get even” that in turn may motivate employees to engage in workplace deviance behavior (Bordia et al., 2008).

The psychological contract also contains expectations concerning “acceptable” workplace conditions and social norms at the workplace (Salin and Notelaers, 2017). Employees are likely to expect that their employer provides a safe work environment and that they will be treated with respect and dignity. However, when an employee becomes the target of permanent negative acts, this expectation would certainly be violated (Salin and Notelaers, 2017). As a consequence of these violations of expected social norms at the workplace, targets of bullying will expect the organization to end this mistreatment (Parzefall and Salin, 2010). If the organization fails to react accordingly, this will result in feelings of betrayal in the target of bullying. Therefore, a perceived contract breach that fosters feelings of psychological contract violation may serve as the mechanism through which workplace bullying exposure leads to a negative evaluation of the employment relationship (Parzefall and Salin, 2010) and the associated negative attitudes that come with this evaluation (e.g., lower job satisfaction). In line with this theoretical reasoning, psychological contract breach or violation has been found to mediate the association between workplace bullying exposure and turnover intentions (Salin and Notelaers, 2017), work engagement (Rai and Agarwal, 2017), and job and life satisfaction (Kakarika et al., 2017).

**Hypothesis 1:** Psychological contract violation mediates the effects of workplace bullying on well-being, job satisfaction, vigor, subjective work performance, burnout, workplace deviance, and turnover intentions.

### Workplace Bullying and Basic Psychological Needs

A complementary approach to explain the link between workplace bullying exposure and work-related attitudes and behavior draws on SDT (e.g., Deci and Ryan, 2000). Based on a large number of empirical studies, SDT assumes that autonomy, competence, and relatedness constitute the three basic human psychological needs that have to be satisfied in order to achieve optimal functioning in individuals. *Autonomy* refers to the individual's experience of freedom, volition, and self-endorsement of choices and action, as well as the absence of salient external controls (Ryan, 1995). *Competence* refers to the individual's need to express his/her capabilities, to master his/her environment, and to experience optimal challenges and positive feedback (Ryan, 1995). Finally, *relatedness* refers to the need of belongingness and connectedness to others and the feeling of being cared of and having significant relationships (Baumeister and Leary, 1995). In the context of work, research has linked need satisfaction to various psychological health indicators such as general well-being, engagement, burnout, and job satisfaction (Van den Broeck et al., 2016). Furthermore, as low need satisfaction reduces engagement, it is not surprising that it has also been associated with lower work performance (Baard et al., 2004). Moreover, reduced job satisfaction correlates with higher turnover intentions (Van den Broeck et al., 2016). Finally, basic need satisfaction was found to be related to deviant workplace behavior (Lian et al., 2012).

As workplace bullying exposure appears to be one of the most serious social stressors (Nielsen and Einarsen, 2012), it has the potential to thwart all three outlined basic psychological needs (Aquino and Thau, 2009). For example, one form of workplace bullying exposure manifests itself through excessive controlling behavior that aims at restricting the target's freedom, volition, and self-endorsement of choices and actions (e.g., unreasonable deadlines, excessive monitoring of one's work). For employees, these negative acts may lead to feelings of constraint and repression, thereby undermining the need for autonomy (Trépanier et al., 2016). Furthermore, perpetrators of workplace bullying may also aim at cutting down the targets accomplishments (e.g., persistent criticism) or taking the target “out of the game” (e.g., removing key areas of responsibility). These kinds of negative behaviors likely thwart employee's need for competence (Trépanier et al., 2016). Finally, workplace bullying behavior may aim at isolating and ostracizing the bullying target (e.g., being excluded from meetings). These forms of negative acts may frustrate the affected employee's need for relatedness (Trépanier et al., 2013). In summary, we can hypothesize that compared to psychological contract violation, frustration of basic needs constitutes a complementary mechanism through which workplace bullying exposure leads to detrimental effects on the target's health, work-related attitudes, and behavior.

**Figure 1:**
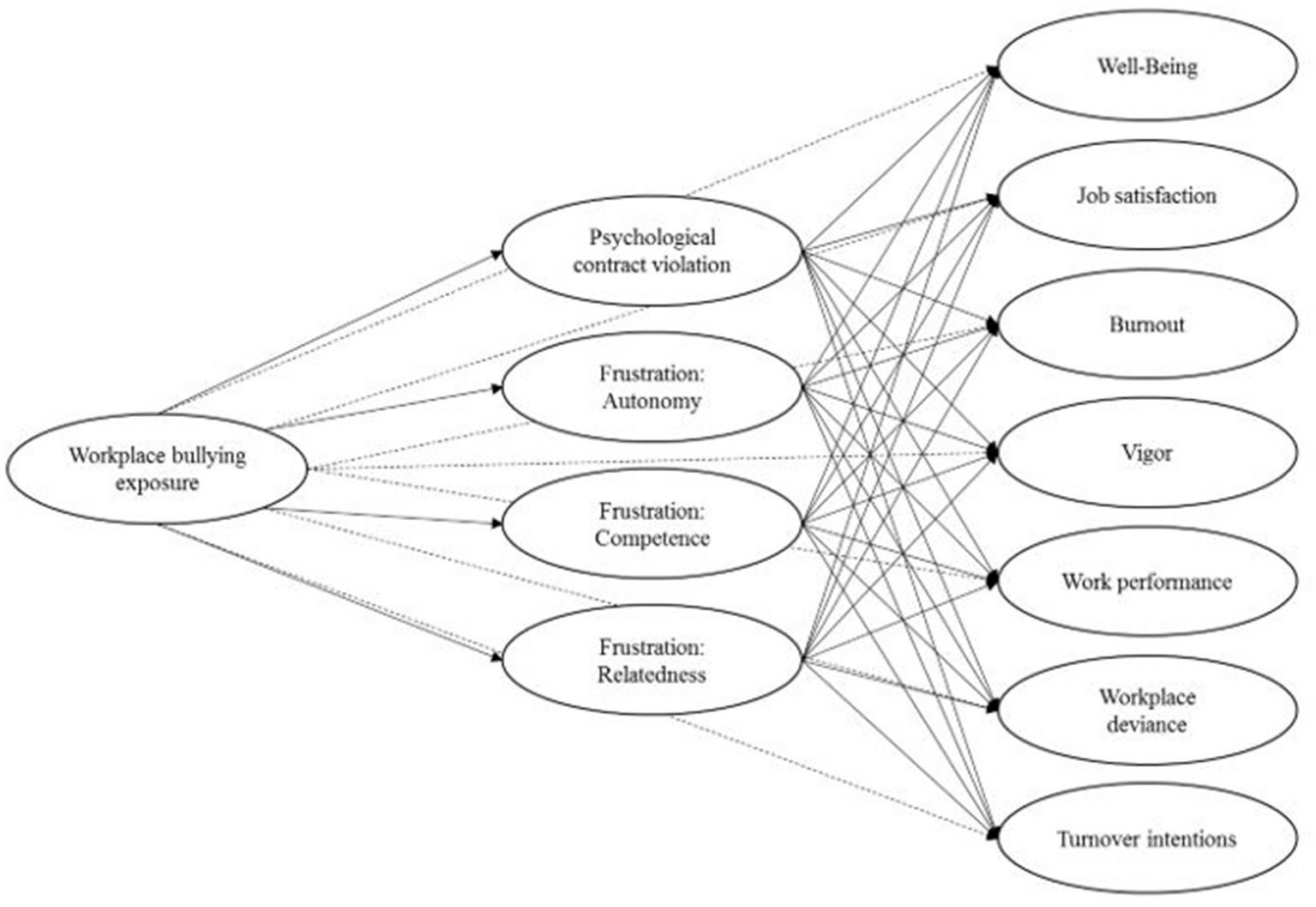
The proposed model. Solid lines, hypothesized associations; dashed lines, controlled associations.

Indeed, decreased basic need satisfaction or increased need frustration has been found to mediate the association between workplace bullying exposure and burnout, work engagement, turnover intentions, psychosomatic complaints, and life satisfaction (Trépanier et al., 2013, 2015, 2016; Goodboy et al., 2020). In SDT, none of the needs is thought to be more important than the others (Van den Broeck et al., 2016). This led some scholars to assess basic need satisfaction or frustration with an overall composite measure rather than each psychological need separately (e.g., Trépanier et al., 2015). However, a recent meta-analysis (Van den Broeck et al., 2016) showed that the different needs incrementally predict differential outcomes. Therefore, in contrast to recent research, we conceptualize the needs as three correlated factors, rather than as one factor that represents an overall need frustration score. Figure 1 presents the developed model.

**Hypotheses 2–4:** Frustration of employee's need for autonomy (H2), competence (H3), and relatedness (H4) incrementally mediates the effects of workplace bullying on well-being, job satisfaction, vigor, subjective work performance, burnout, workplace deviance, and turnover intentions.

## Methods

### Participants and Procedure

To test our hypotheses, we recruited participants *via* Amazon's Mechanical Turk (MTurk; Buhrmester et al., 2011; Crump et al., 2013) because it offers an opportunity for examining a wider range of occupations (Keith et al., 2017). Research has shown that many behavioral occupational health–related associations show comparable effect sizes as in published benchmarks (e.g., Michel et al., 2018). We followed recent recommendations to strengthen validity inferences using MTurk as participant recruiting system (Cheung et al., 2017; Keith et al., 2017), e.g., prescreening of the target population, fair payment (i.e., US $0.10 per estimated minute of participation; Chandler and Shapiro, 2016), and data screening methods for insufficient effort responding (McGonagle et al., 2016). For data collection purposes, we used the TurkPrime platform (Litman et al., 2017) that allows to verify workers' country location and block individuals trying to participate more than one time using identical Internet Protocol addresses. Workers who were employed and reside in the United States were invited for the prescreening 10-item online survey (named as demographic survey) and compensated with US $0.10^2^. We sampled 4,014 respondents (59.3% females, *n* = 2,378). Completion rate (percentage of workers who started and finished the survey) was high (97.5%), whereas bounce rate (percentage of workers who previewed a survey and did not accept it) was low (9.3%), indicating a low level of self-selection (Keith et al., 2017). Two weeks later, we invited those workers who matched our inclusion criteria (at least part-time employed and working with supervisors and colleagues: 54.3%, *n* = 2,179) to participate in a “working condition survey” (estimated duration of 12 min)^3^. A total of 1,609 participants (73.8%) took part and were compensated each with US $1.20.^4^ No forced answering design was implemented as this has been found to be detrimental in terms of data quality (Sischka et al., 2020b). We filtered out workers who indicated that their employment status had changed between prescreening and the actual survey (e.g., from employment to unemployment; 0.9%; *n* = 15). Furthermore, some respondents were excluded because of missing data (1.7%, *n* = 27). Respondent's median completion time was 11.4 min.

In order to increase data quality, we screened the data for insufficient effort responding (McGonagle et al., 2016). Therefore, we included two attention check (i.e., instructed response) items, used a time screen, and implemented self-report questions about data quality at the end of the questionnaire. As time screening instrument, we used the response time indicator second per item (spi) provided by (Wood, 2017). The four self-report questions (DeSimone and Harms, 2018) inquiring respondents to indicate the frequency of answering questions honestly (reverse-scored), responding without carefully reading the questions, putting thought into survey responses (reverse-scored), and using little effort when selecting answers to ensure data quality. The response format for the self-report questions ranged from 1 (totally disagree) to 7 (totally agree), with higher scores indicating potential insufficient effort responding. We excluded respondents who failed to correctly answer one or both instructed response items and/or or had an spi < 1 per item block more than once and/or scored above 3 (disagree somewhat) on the average self-reported data quality items (19.3%, *n* = 310) from further analysis. Thus, the final sample contained 1,257 respondents (57.4% females, *n* = 722), with ages ranging from 20 to 73 (mean = 37.7, SD = 10.5) and organization tenure ranging from less than a year to 51 years (mean = 6.3, SD = 6.7). Most respondents had a permanent work contract (87.1%, *n* = 1,095). The majority of respondents were Caucasian (80.8%).

### Measures

#### Workplace Bullying Exposure

We used the nine-item Short-Negative Acts Questionnaire (S-NAQ; Notelaers et al., 2019) to assess exposure to workplace bullying. Respondents indicated how frequently they have been exposed to each of these negative acts (e.g., “Someone withholding information which affects your performance”) on a scale from 1 (never) to 5 (always). We did not include a time frame as some studies questioned the frequently applied 6-month criterion (see the discussion in Sischka, 2018). Especially it has been criticized that there is a lack of empirical evidence regarding the 6-month criterion (Vranjes et al., 2017). Moreover, a study investigating physiological stress response to workplace bullying exposure found an association between bullying frequency and the amount of salivary cortisol, but no association between bullying duration and amount of salivary cortisol (Hansen et al., 2011). Furthermore, semi-structured interviews with working professionals revealed that they regarded the 6-month criterion as too long and that the targeted employees showed serious stress reactions within much shorter time frames (e.g., within 1 month; Vranjes et al., 2017).

#### Psychological Contract Violation

To measure psychological contract violation, we used the four-item scale from Robinson and Morrison (2000) (e.g., “I feel betrayed by my organization”). The response format ranged from 1 (totally disagree) to 7 (totally agree).

#### Basic Psychological Need Frustration

We used the Psychological Needs Thwarting Scale (Bartholomew et al., 2011) that was modified to fit the work context (see also Trépanier et al., 2016; Olafsen et al., 2017). This scale assesses the frustration of the need for autonomy (four items; e.g., “I feel prevented from making choices with regard to the way I do my work”), competence (four items; e.g., “There are times at work when I am told things that make me feel incompetent”), and relatedness (four items; e.g., “At work, I feel other people dislike me”). All items had a response format ranging from 1 (totally disagree) to 7 (totally agree).

#### Well-Being

The five-item WHO-5 Well-Being Index is a well-validated brief general index of subjective psychological well-being (Topp et al., 2015; Sischka et al., 2020a) with responses ranging from 1 (at no time) to 6 (all of the time). A sample item is “Over the past 2 weeks, I have felt cheerful and in good spirits.”

#### Job Satisfaction

We used the three-item Michigan Organizational Assessment Questionnaire Job Satisfaction Subscale (Cammann et al., 1983). A sample item is “All in all I am satisfied with my job.” The response format ranged from 1 (totally disagree) to 7 (totally agree).

#### Burnout

We used the seven-item work-related burnout subscale of the Copenhagen Burnout Inventory (Kristensen et al., 2005). A sample item is “Do you feel that every working hour is tiring for you?” The response scale ranged from 1 (never) to 5 (always).

#### Vigor

The three-item vigor subscale of the Utrecht Work Engagement Scale (Schaufeli et al., 2006) is characterized by high levels of energy and the willingness to invest effort in one's work, even when it comes to difficulties and problems. Vigor was included as it represents the direct opposite of the core burnout dimension of exhaustion (González-Romá et al., 2006) that is assessed with the Copenhagen Burnout Inventory. Thus, the full continuum of employee's energy and mental resilience was captured. A sample item is “At my work, I feel bursting with energy.” Response alternatives ranged from 1 (totally disagree) to 7 (totally agree).

#### Work Performance

Subjective work performance was assessed by two items (Sischka et al., 2020d), including “How do you rate your overall work performance compared to your colleagues?” and “How does your supervisor rate your overall work performance?” Participant responded on a seven-point scale ranging from 1 (far below average) to 7 (far above average).

#### Workplace Deviance

We used five items of the organizational deviance scale from Bennett and Robinson (2000) (7-point response scale ranging from 1 = totally disagree to 7 = totally agree). A sample item is “Put little effort into your work.”

#### Turnover Intentions

We used the three-item scale of Sjöberg and Sverke (2000). A sample item is “I am actively looking for other jobs.” Participants responded on a seven-point scale ranging from 1 (totally disagree) to 7 (totally agree).

### Statistical Analyses

Given that the distribution of indicators has a strong influence on confirmatory factor analyses (CFAs) and structural equation modeling (SEM) estimation results, univariate, and multivariate distributions of the items were analyzed. Subsequently, we tested the proposed measurement model with CFA in order to guarantee construct validity. The MLR χ^2^-test statistic (Yuan and Bentler, 2000) and respective fit indices were calculated as they provide more accurate estimations for items with five or more answer categories and for distortion from univariate and multivariate normality (Finney and DiStefano, 2013). We also implemented the unmeasured latent method construct procedure in order to check for the existents and extent of common method variance (CMV) that might be an alternative explanation for the correlations of our substantive variables (Williams and McGonagle, 2016)^5^. Next, we examined reliability, (latent) means, standard deviations, and zero-order correlations between the constructs within CFA. As the most popular measure of reliability, Cronbach's α, has some deficiencies (i.e., relying on assumptions that are very strict and unrealistic), we instead calculated McDonald's ω (McDonald et al., 1999) as a measure of internal consistency that makes fewer and more realistic assumptions (Dunn et al., 2014). The effects-coding method was used for scale setting to estimate each construct's latent mean and variance in a non-arbitrary metric (Little et al., 2006) so that the latent constructs have a theoretical range similar to the manifest items. Furthermore, we conducted analyses of zero-order correlations to get a first impression of the associations between constructs utilizing phantom constructs in order to calculate the covariance between the latent variables in correlational metric (Little, 2013).

In a next step, we tested a multiple mediator model within an SEM approach to evaluate the individual influence of feelings of psychological contract violation and basic need frustration by controlling for possible multicollinearity (MacKinnon et al., 2012). Point and interval estimators for the standardized indirect effects were calculated. To obtain the 95% confidence intervals (CI95), the percentile bootstrap approach was applied (Davison and Hinkley, 1997) as it has a good coverage probability for obtaining confidence intervals for the indirect effect in standardized metric (Cheung, 2009) in SEM framework (we drew 10,000 bootstrap samples). R version 3.6.0 (R Core Team, 2019) was used for data analysis.

## Results

### Factor Analysis

As subjective work performance contained only two indicators, their factor loadings were set equal in order to avoid estimation problems and improper solutions (e.g., Heywood cases). Table 1 shows the CFA results. Other competing measurement models were tested in order to guarantee that the study's constructs were distinct. Table 1 shows that the expected 12-factor solution fitted the data better than a 10-factor (workplace bullying exposure, psychological contract violation, frustration autonomy, frustration competence + frustration relatedness, well-being, burnout, vigor, job satisfaction + turnover intentions, workplace deviance, and work performance), 11-factor (additionally including turnover intentions), or 13-factor solution (like the 12-factor model but with a second-order factor for basic need frustration). Adding a method factor (Method_U_) to the 12-factor model further increased model fit. Restricting the method factor loadings to be equal within substantive latent constructs (Method_I_) decreased the model fit. Thus, we used Method_U_ as reference model. Within this model, we fixed factor correlations of the substantive latent constructs to values from model 5 (Method-R) with the result of increased model fit. Therefore, one might conclude that although some CMV exists, it exerted no substantial influence on the substantive interrelations. Thus, we continued the analyses with 12 latent factors.

**Table 1:** Fit statistics for different measurement models.

**#**	**Model**	**χ^2^**	***df***	***P***	**RMSEA [CI_**90**_]**	**CFI**	**TLI**	**SRMR**	**AIC**	**BIC**
1.	Single factor	19,776.664	1,326	0.000	0.105 [0.104–0.106]	0.545	0.527	0.101	197,195	197,735
2.	9 Factors	6,075.430	1,290	0.000	0.054 [0.053–0.056]	0.882	0.874	0.053	179,798	180,523
3.	10 Factors	5,846.983	1,281	0.000	0.053 [0.052–0.054]	0.887	0.879	0.054	179,530	180,301
4.	11 Factors	5,344.883	1,271	0.000	0.050 [0.049–0.052]	0.899	0.891	0.053	178,927	179,749
5.	12 Factors	5,023.273	1,260	0.000	0.049 [0.047–0.050]	0.907	0.898	0.052	178,541	179,419
6.	13 Factors^a^	5,146.961	1,278	0.000	0.049 [0.048–0.050]	0.904	0.897	0.053	178,657	179,443
7.	12 Factors (Method_U_)	4,173.173	1,195	0.000	0.045 [0.043–0.046]	0.926	0.915	0.035	177,602	178,814
8.	12 Factors (Method_I_)	4,730.047	1,236	0.000	0.047 [0.046–0.049]	0.914	0.904	0.051	178,147	179,149
9.	12 Factors (Method-R)	3,997.284	1,261	0.000	0.042 [0.040–0.043]	0.932	0.926	0.041	177,233	178,106

*MLR estimator; RMSEA [CI90], root mean squared error of root mean squared error of approximation with 90% confidence intervals; CFI, comparative fit index; TLI, TuckerLewis index; SRMR, standardized root mean square residual; AIC, Akaike information criterion; BIC, Bayesian information criterion*.

a *Basic need frustration as second-order factor. Method_U_, 12 factors + inclusion of method factor; Method_I_, 12 factors + inclusion of method factor with equal method factor loadings within and freely estimated method factor loadings between substantive latent constructs; Method-R, 12 factors + inclusion of method factor but with restricted correlations of substantive latent constructs from model 5*.

### Descriptives, Correlations, and Reliabilities

Table 2 shows the latent means, standard deviations, and intercorrelations between the study variables and internal consistencies. The correlation analyses offered a first insight into the hypothesized relationships among the constructs. As expected, workplace bullying exposure was highly correlated with feelings of psychological contract violation and frustration of basic needs, especially regarding need for relatedness. Furthermore, workplace bullying exposure was negatively correlated with well-being, job satisfaction, vigor, and work performance. In contrast, workplace bullying exposure was positively correlated with burnout, workplace deviance, and turnover intentions. Finally, feelings of psychological contract violation and frustration of autonomy, competence, and relatedness were negatively associated with well-being, job satisfaction, vigor, and subjective work performance. Positive relations were found between feelings of psychological contract violation and burnout, workplace deviance, and turnover intentions. Notably, feelings of psychological contract violation and any indicator of basic need frustration were substantially positively associated.

**Table 2:** Latent means, standard deviations, intercorrelations, and reliabilities.

		**Mean**	**SD**	**1**.	**2**.	**3**.	**4**.	**5**.	**6**.	**7**.	**8**.	**9**.	**10**.	**11**.	**12**.
1.	Workplace bullying	1.69	0.65	**0.90**											
				**[0.89** to **0.91]**											
2.	Psychological contract violation	2.28	1.52	0.62	**0.95**										
				[0.58 to 0.67]	**[0.95** to **0.96]**										
3.	Frustration: autonomy	3.57	1.29	0.62	0.62	**0.83**									
				[0.57 to 0.67]	[0.58 to 0.67]	**[0.81** to **0.85]**									
4.	Frustration: competence	2.92	1.43	0.70	0.63	0.85	**0.88**								
				[0.66 to 0.75]	[0.58 to 0.68]	[0.81 to 0.89]	**[0.86** to **0.89]**								
5.	Frustration: relatedness	2.77	1.23	0.80	0.67	0.80	0.86	**0.83**							
				[0.76 to 0.84]	[0.62 to 0.71]	[0.76 to 0.84]	[0.82 to 0.90]	**[0.81** to **0.85]**							
6.	Well-being	3.74	1.05	−0.38	−0.49	−0.50	−0.50	−0.52	**0.91**						
				[−0.44 to −0.32]	[−0.54 to −0.44]	[−0.56 to −0.45]	[−0.55 to −0.45]	[−0.57 to −0.47]	**[0.90** to **0.92]**						
7.	Job satisfaction	4.97	1.57	−0.47	−0.74	−0.62	−0.59	−0.59	0.65	**0.94**					
				[−0.52 to −0.41]	[−0.77 to −0.70]	[−0.67 to −0.57]	[−0.64 to −0.55]	[−0.64 to −0.54]	[0.61 to 0.69]	**[0.93** to **0.95]**					
8.	Burnout	3.14	0.86	0.54	0.60	0.62	0.61	0.58	−0.63	−0.70	**0.92**				
				[0.49 to 0.59]	[0.56 to 0.65]	[0.57 to 0.67]	[0.57 to 0.66]	[0.53 to 0.63]	[−0.68 to −0.59]	[−0.74 to −0.67]	**[0.91** to **0.93]**				
9.	Vigor	3.77	1.49	−0.37	−0.51	−0.52	−0.52	−0.54	0.76	0.75	−0.71	**0.91**			
				[−0.43 to −0.32]	[−0.55 to −0.46]	[−0.58 to −0.47]	[−0.57 to −0.47]	[−0.59 to −0.49]	[0.72 to 0.79]	[0.72 to 0.78]	[−0.75 to −0.67]	**[0.90** to **0.92]**			
10.	Work performance	5.28	0.85	−0.16	−0.20	−0.30	−0.39	−0.31	0.32	0.29	−0.19	0.35	**0.79**		
				[−0.23 to −0.09]	[−0.27 to −0.13]	[−0.36 to −0.23]	[−0.46 to −0.33]	[−0.38 to −0.24]	[0.26 to 0.38]	[0.22 to 0.36]	[−0.26 to −0.12]	[0.29 to 0.41]	**[0.75** to **0.82]**		
11.	Workplace deviance	1.79	0.62	0.26	0.29	0.39	0.38	0.36	−0.35	−0.38	0.39	−0.50	−0.29	**0.82**	
				[0.20 to 0.33]	[0.22 to 0.35]	[0.33 to 0.45]	[0.32 to 0.45]	[0.30 to 0.43]	[−0.41 to −0.29]	[−0.44 to −0.32]	[0.33 to 0.44]	[−0.55 to −0.44]	[−0.36 to −0.22]	**[0.80** to **0.83]**	
12.	Turnover intentions	3.59	1.68	0.41	0.62	0.55	0.51	0.51	−0.49	−0.82	0.59	−0.61	−0.16	0.33	**0.87**
				[0.36 to 0.46]	[0.57 to 0.66]	[0.49 to 0.60]	[0.46 to 0.56]	[0.46 to 0.55]	[−0.54 to −0.44]	[−0.85 to −0.80]	[0.55 to 0.64]	[−0.66 to −0.57]	[−0.23 to −0.09]	[0.27 to 0.39]	**[0.85** to **0.88]**

*Coefficients display zero-order correlations and in parentheses CI_95_; internal consistencies (McDonald's ω) in the main diagonal (bold face, CI95 in parentheses)*.

### Multiple Mediation Analysis

**Figure 2:**
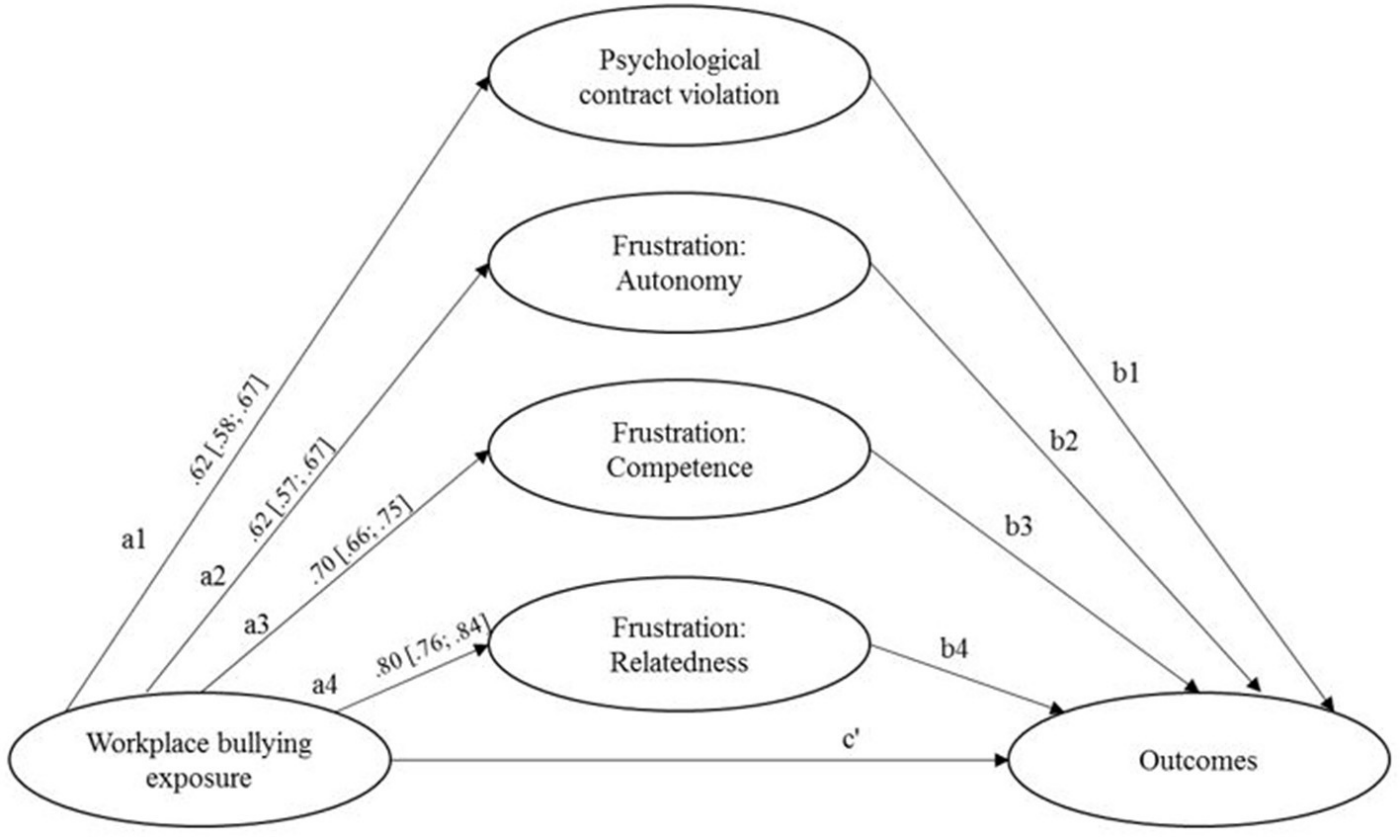
Psychological contract violation and basic need frustrations as mediators between workspace bullying and outcomes. Standardized effects. Covariance among independent variables, item-level structure of the constructs, error terms, and correlations between error terms of dependent variables are not shown, for simplicity and clarity. CI_95_ based on 10,000 bootstrap samples calculated with percentile bootstrap approach; $R_{\text{Psychological contract violation }=0.39;}^{2}$ $R_{\text{Frustration autonomy }=0.39}^{2}$; $R_{\text{Frustration Competence }=0.50}^{2}$; $R_{\text{Frustration Relatedness }=0.64}^{2}$.

**Figure 3:**
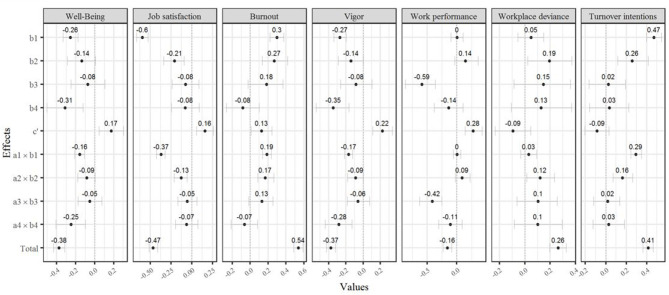
Psychological contract violation and basic need frustrations as mediators between workspace bullying and outcomes: Parameters. For paths related to “a” see Figure 2. CI_95_ based on 10,000 bootstrap approach; $R_{\text{well}-\text{being}}^{2}$ = 0.33; $R_{\text{job satisfaction}}^{2}$ = 0.59; $R_{\text{burnout}}^{2}$ = 0.447; $R_{\text{vigor}}^{2}$ = 0.36; $R_{\text{work performance}}^{2}$ = 0.19; $R_{\text{workplace deviance}}^{2}$ = 0.17; $R_{\text{turnover intentions}}^{2}$ = 0.42.

In order to identify the independent contributions and the most powerful mediators for the explanation of the different outcomes, we tested a model that included all mediators concurrently^6^. Figure 2 shows the specified structural model. All outcome variables were included in this model with correlated error terms. This model showed an acceptable fit to the data [χ^2^ = 5,023.273, *df* = 1,260, *p* <0.001, root mean squared error of approximation (RMSEA) [CI_90_] = 0.049 [.047;0.050], standardized root mean square residual (SRMR) = 0.051, comparative fit index (CFI) = 0.907, Tucker–Lewis index (TLI) = 0.898]. As the inspection of the correlational analysis already suggested, the variance inflation factors (VIF) indicated that the mediators were multicollinear (VIF_violation_ = 1.88, VIF_autonomy_ = 3.93, VIF_competence_ = 5.37, VIF_relatedness_ = 4.53). However, the variance inflation factors fell below the suggested cutoff value for extreme multivariate collinearity of VIF > 10 (Kline, 2016), thus allowing for estimation of the effects of all these variables. For well-being, psychological contract violation and frustration of relatedness served as substantial mediators (Figure 3). Regarding job satisfaction, burnout and vigor, psychological contract violation, and frustration of autonomy mediated the paths between workplace bullying and these outcomes. For vigor, however, frustration of relatedness was the strongest mediator. Frustration of competence was the only significant predictor of work performance. Furthermore, for workplace deviance, only the frustration of autonomy had a significant mediation effect. Regarding turnover intentions, psychological contract violation, and frustration of autonomy turned out to significantly mediate the relation between workplace bullying exposure and this outcome variable.

## Discussion

The present study provides detailed insights into the psychological mechanisms underlying differential effects of workplace bullying exposure on a number of variables that capture health, work-related attitudes, and workplace behavior (i.e., well-being, job satisfaction, burnout, vigor, work performance, workplace deviance, turnover intentions). Multiple mediation analyses allowed an assessment of the specific mediating effect of each variable tested, conditional on the presence of other mediators in the model.

Based on this method, different mediators were identified indicating psychological mechanisms that link workplace bullying exposure and its negative consequences. We replicated previous findings that feelings of psychological contract violation denote a psychological mechanism that contributes to the link between workplace bullying exposure and work engagement (Rai and Agarwal, 2017), as well as the link between workplace bullying exposure and turnover intentions (Salin and Notelaers, 2017). However, as Salin and Notelaers (2017) suggested, other processes also affect turnover intentions. In addition to psychological contract violation, frustration of the need for autonomy was also found to mediate the effect of workplace bullying on turnover intentions. Similarly, frustrating both the need for autonomy and relatedness mediated the relation between bullying and vigor. Therefore, the present findings are also consistent with previous studies on workplace bullying exposure and basic psychological needs (Trépanier et al., 2013, 2015, 2016). At the same time, we also extended these studies by simultaneously testing both psychological mechanisms. Therefore, we were able to calculate the individual effect of each mediator net of the others. Furthermore, we showed that feelings of psychological contract violation also play an important role as mediator between bullying exposure and both job satisfaction and burnout. We also explored the link between bullying and subjective work performance and found that frustrating competence appears to be even more important (i.e., detrimental) than feelings of psychological contract violation.

### Theoretical Implications

The results of the present study showed that being on the receiving end of constant negative behavior violates the expectations of a safe work environment where one expects to be treated with respect and dignity. In line with affective events theory (Weiss and Cropanzano, 1996), feelings of contract violation are associated with lower levels of well-being and higher levels of burnout, as permanent negative emotions have an impact on employees' psychological health. Furthermore, concomitant with social exchange theory that emphasizes the importance of reciprocity to understand the evaluation of one's relation with other parties (Cropanzano and Mitchell, 2005), feelings of contract violation render the job less valuable to the employee. Consequences are lower job satisfaction, lower vigor, and higher turnover intentions.

Regarding the explanatory contributions of each basic need, and in line with a recent review and meta-analysis of SDT (Van den Broeck et al., 2016), autonomy is the strongest predictor for job satisfaction, burnout, and turnover intentions, whereas competence appears to be the most important factor for work performance. In contrast to the meta-analysis, relatedness, but not competence and autonomy, is the strongest predictor for well-being and vigor. However, in opposition to most previous studies referring to SDT, we directly studied need frustration in contrast to need satisfaction, as this is a more direct test of our hypothesis regarding the mediating process of SDT in the context of workplace bullying. As Vansteenkiste and Ryan (2013) pointed out, need frustration may relate more robustly to malfunctioning than low need satisfaction. Therefore, different patterns may occur when one studies need frustration compared to need satisfaction. In contrast to the meta-analysis by Van den Broeck et al. (2016), the present study found that relatedness is more important for well-being and vigor than competence and autonomy. This may be due to the fact that frustration of relatedness may display feelings of ostracism and isolation and therefore may have a stronger relation to well-being. In contrast, low satisfaction of relatedness (at work) can be easily compensated with relationships outside of the work context (i.e., family, friends). In this regard, low need satisfaction may be just the absence of work-related friendships. This is also supported by Trépanier et al. (2016), who simultaneously studied the longitudinal influence of basic need satisfaction and frustration. While frustration of relatedness was linked with decreased life satisfaction 1 year later, relatedness satisfaction was not. The need for belongingness is a fundamental human need that, if unfulfilled, may have detrimental effects when a certain threshold is reached (Baumeister and Leary, 1995).

The results of the present study also revealed that feelings of psychological contract violation and frustration of basic needs each accounted for unique variation in well-being, job satisfaction, burnout, vigor, and turnover intentions, thus pointing to the individual contribution of both psychological mechanisms. However, when controlled for frustration of basic needs, feelings of psychological contract violation were no longer related to work performance. Therefore, feelings of psychological contract violation seem only spuriously correlated with work performance, which may be explained by the association of frustration of competence and work performance. Furthermore, when controlled for frustration of basic needs, feelings of psychological contract violation were also no longer related to workplace deviance.

### Practical Implications

Effective interventions depend on specific knowledge about “the mechanisms that can explain the detrimental effects of bullying” (Nielsen and Einarsen, 2018, p. 79). Thus, the results of this study may offer pertinent implications for practitioners on all levels of preventive measures. The current findings can guide possible theory-based primary-, secondary-, and tertiary-stage interventions on organizational as well as individual levels that aim to prevent, reverse, or buffer the negative progression of the bullying incident; help the target to cope with the situation; and restore their health and trust in the organization (Zapf and Vartia, 2020).

Primary-stage intervention might focus on potential risk factors of workplace bullying, for instance, on leadership, organizational climate, and the work environment. Employers can train their supervisors to adopt a leadership style that takes employees' individual basic needs into consideration, for instance, with a transformational leadership style (Hetland et al., 2011) that has been shown to be associated with lower levels of workplace bullying exposure (Nielsen, 2013). Moreover, an ethical infrastructure—formal and informal systems in an organization that facilitate ethical and inhibit unethical behavior—has been shown to be related to successful handling of workplace bullying incidents (Einarsen et al., 2017). Organizations may also redesign certain job characteristics to prevent bullying incidents as certain characteristics (e.g., higher job demands, lower job autonomy) are associated with higher workplace bullying exposure (Li et al., 2019).

Secondary-stage interventions aim to prevent the escalation of conflicts that might ultimately trigger bullying incidents (Zapf and Vartia, 2020). Workplace bullying in its early stages might be counteracted with various organizational conflict management procedures (e.g., counseling, moderation, mediation). These measures might disrupt the effect of bullying exposure on psychological contract violation if they are perceived as fair and impartial (Zapf and Vartia, 2020).

Finally, helping employees to deal effectively with the bullying incident (e.g., complaints procedures, conflict arbitration, support in finding counseling or therapy, stress management training) should buffer the negative effect of workplace bullying exposure (tertiary-stage intervention; Zapf and Vartia, 2020) and reduce their experienced frustration of basic needs, preserving their well-being, vigor, and work performance, and, eventually, prevent burnout. These interventions might restore employee's feeling of trust in the organization and prevent or reduce feelings of psychological contract violation. On the other hand, “just wait and see” without taking action is likely to have detrimental consequences. Without organizational measures that aim at preventing workplace bullying or with measures that do not function adequately, the target may ultimately blame the organization for their situation. This attribution process may increase feelings of psychological contract violation, resulting in lower job satisfaction and even turnover intentions. Therefore, the employees should experience that someone in the organization takes care of their situation and will take appropriate steps against workplace bullying.

## Limitations and Future Research

Some limitations of the present study need to be considered that provide directions for future research. First, our data are correlational, thus lacking time precedence, and the predicted mediations were only theory-driven (e.g., Trépanier et al., 2013; Salin and Notelaers, 2017). Any causal assumptions cannot be drawn. Note, however, that the tested model is consistent with empirical results of experimental (Kakarika et al., 2017) and longitudinal studies on psychological contract breach and violation (e.g., Bordia et al., 2008) and longitudinal studies on basic psychological needs (e.g., Trépanier et al., 2016). Particularly, Trépanier et al. (2016) have already shown the longitudinal effects of workplace bullying exposure on basic need frustration. Nevertheless, future research should apply longitudinal designs that will also provide information on the development of the different effects over time. Second, an additional limitation is the mono-method design, as only self-reported measures were employed. Although we have statistically controlled for CMV, we cannot fully rule out the possibility that this may have led to an overrating of the effects (Podsakoff et al., 2012). However, Conway and Lance (2010) stated that under certain conditions, self-report can be acceptable or even necessary, especially when there is evidence of construct validity, a lack of overlap in items for different constructs, and when tested for CMV. In the present study, the CFAs revealed that the proposed 12-factor model showed the best fit to the data. This substantiates the construct validity and the absence of larger amounts of item overlapping as respondents were reasonably able to conceptually distinguish between constructs. Furthermore, many constructs such as psychological contract violation, basic need frustration, or job satisfaction are necessarily subjective, which renders self-reports appropriate and even necessary (Conway and Lance, 2010). In contrast, this is not necessarily the case for the measures of work motivation, work performance, and workplace deviance that may suffer from greater influence by social desirability. Therefore, analyses for these outcome variables need to be seen more critically. Future research should utilize multiple, preferably behavioral data sources. Third, a lack of generalizability of the findings may result from the convenience sample in the MTurk approach. However, compared to other convenient sampling strategies, MTurk has the advantage of providing easy access to a more heterogeneous employment population. Therefore, findings are not just limited to only one type of industry. This makes MTurk ideal for testing organizational theories expected to be broadly applicable across different organizational settings (Cheung et al., 2017) as it is the case in the present study. Nevertheless, future studies should test the proposed model in other working populations and with other samples. Fourth, multicollinearity between predictors or mediators (as was found in the present study) has the potential to inflate type II error rates (Grewal et al., 2004). However, given the high reliability of our measures (ω between 0.79 and 0.95) and the large sample size that counter multicollinearity effects (Grewal et al., 2004), we are confident that our estimates are sufficiently accurate. Fifth, because of time and space constraints, only the short version (S-NAQ) of the negative acts questionnaire was used. Future studies might utilize the long version of this questionnaire (Einarsen et al., 2009) to capture a wider range of negative acts at work. Nevertheless, the S-NAQ has been shown to have high construct validity (Notelaers et al., 2019). A final limitation concerns our sample that consisted of predominantly Caucasian participants.

Future studies might seek to investigate possible moderation effects between workplace bullying exposure and psychological contract violation, as well as between workplace bullying exposure and frustration of basic needs. The target's perception and attribution of the bullying incidents have an impact on the emotional experience (Oh and Farh, 2017) and thus on perceived psychological contract breach and feelings of violation. Especially, when the target attributes the bullying exposure to himself/herself, he/she should not perceive a psychological contract violation. However, the attribution process might be influenced by the microcontextual characteristics of the negative acts (Nishina and Bellmore, 2010). For instance, more subtle bullying behavior might be less likely perceived as bullying behavior and will rather lead to confusion (Samnani et al., 2013) and to self-attribution of the target (Bowling and Beehr, 2006). Furthermore, situations with many perpetrators and passive bystanders may also be more likely to elicit self-attribution (Nishina, 2012). Moderators between workplace bullying exposure and frustration of basic needs may include individual dispositions of the targets. For instance, hardiness describes “a person's predisposition to be resistant to the harmful effects of stressors and effectively adapt and cope with a demanding environment” (Eschleman et al., 2010, p. 277). Hardy people believe that they are able to control experienced events, perceive difficult situations as challenges rather than threats, and are self-committed (Delahaij et al., 2010). Indeed, Reknes et al. (2018) found that hardiness was a strong moderator between the workplace bullying exposure and mental health association, in that hardy employees did not experience increased levels of anxiety with increased bullying exposure.

Future studies might also research possible conditional effects that buffer or exacerbate the effects of psychological contract violation and basic need frustration on different outcomes. Feelings of psychological contract violation might lead to revenge cognitions that might translate into lower work performance and deviant behavior, especially when self-control of the respective person is low (Bordia et al., 2008). The negative behavioral reaction of an employee (e.g., lower work performance, more deviant behavior) who experiences feelings of psychological contract violation and in turn revenge cognitions might also be moderated by fear toward the perpetrator (Marcus-Newhall et al., 2000). Moreover, traditional SDT scholars (e.g., Deci and Ryan, 2000) have seen needs as innate and universal, thus focusing their research on need *satisfaction* or *frustration* rather than individual's need *strength* (Van den Broeck et al., 2016). However, this view has been challenged (e.g., Van Assche et al., 2018). Therefore, one could hypothesize that the mediation effect of basic need frustration that links workplace bullying with several detrimental outcomes might be moderated by individual need strength.

## Conclusion

The present study furthers the understanding of psychological mechanisms that underlie the relation between workplace bullying exposure and its effects on health, work-related attitude, and behavior. Based on psychological contract theory and SDT, different mediators (i.e., psychological contract violation, frustration of autonomy, competence, relatedness) were identified as important contributing psychological mechanisms. Negative behaviors such as workplace bullying exposure violate the expectations of a safe work environment where one is treated with respect and dignity. As a consequence of these violations of psychological contract and basic needs, negative consequences may occur on the side of the employees.

## Data Availability Statement

The raw data supporting the conclusions of this article will be made available by the authors, without undue reservation.

## Ethics Statement

Ethical review and approval was not required for the study on human participants in accordance with the local legislation and institutional requirements. The patients/participants provided their written informed consent to participate in this study.

## Author Contributions

PS, AM, AS, and GS wrote this manuscript. PS and GS were responsible for research design and ideas and developing the questionnaire. GS, AM, and AS were responsible for research revision. PS was responsible for collecting and analyzing the data. All authors contributed to the article and approved the submitted version.

## Conflict of Interest

The authors declare that the research was conducted in the absence of any commercial or financial relationships that could be construed as a potential conflict of interest.

## Footnotes

^1^We used the term “workplace bullying exposure” to refer to the perspective of the targeted employee who undergoes the experience of being bullied.

^2^Participants were given an informed consent form. The survey was conducted according to the Declaration of Helsinki (i.e., voluntary participation, participants were free to withdraw their consent at any time throughout the interviews without negative consequences for them).

^3^The survey also contained some measures of workplace conditions (see Sischka et al., 2020c).

^4^Gender, age, and ethnicity had some influence on taking the survey (all other demographics *p* > 0.05): Male workers (χ^2^ = 10.534, *df* = 1, *p* < 0.01, Cramer's *V* = 0.07), older workers (*t* = 5.921, *df* = 2,177, *p* < 0.001, *r* = 0.13), and Caucasian, African American, and Asian workers (χ^2^ = 11.282, *df* = 5, *p* < 0.05 Cramer's *V* = 0.07) were more likely to participate in the survey. However, the effect sizes were rather small; therefore, substantial bias in terms of a systematic dropout is highly unlikely.

^5^Therefore, we included three additional CFA models: A model with an additional method factor (Method_U_) to examine whether CMV exists; a model with a method factor with equal method factor loadings within and freely estimated method factor loadings between substantive latent constructs (Method_I_) to check whether the extent of CMV varies between substantive constructs. Of those two models, the model with the better model fit served as the reference model. The third model was based on this reference model but, additionally, with restricted correlations of substantive latent constructs from the last model without the inclusion of a method factor. Bias in substantive relation is indicated if this model shows a substantial deterioration in model fit.

^6^In a first step, we tested psychological contract violation and frustration of basic needs (i.e., frustration of autonomy, competence, and relatedness) in separate mediation models. See the Electronic Supplement for these results.
