# Supplementary material for: Psychological Contract Violation or Basic Need Frustration? Psychological Mechanisms Behind the Effects of Workplace Bullying
Source: Front Psychol. 2021 Apr 9;12:627968. doi: 10.3389/fpsyg.2021.627968 (PMC8062865; doi:10.3389/fpsyg.2021.627968)
Supplement: Supplementary file 1 [file Data_Sheet_1.docx]

**Psychological contract violation or basic need frustration? Psychological mechanisms behind the effects of workplace bullying.**

# Electronical Supplement

## Single mediation analysis

In a first step, we tested whether psychological contract violation mediates the association between workplace bullying exposure and the different outcomes. Figure 1 shows the structural model that included all outcome variables and the specified correlations between their error terms. The model showed a good fit to the data (χ² = 3161.211, *df* = 744, *p* < .001, *RMSEA* [CI_90_] = .051 [.049; .052], *SRMR* = .049, *CFI* = .920, *TLI* = .912). Workplace bullying exposure was a strong predictor for psychological contract violation (Figure 1, path a). Furthermore, psychological contract violation was a significant predictor for all outcome variables (Figure 2, path b), when controlling for the direct effect of workplace bullying exposure (Figure 2, path c’). However, feelings of psychological contract violation had the strongest influence on job satisfaction and turnover intentions. Moreover, the indirect or mediated effect was significant for all outcome variables (Figure 2, path a*b) as well as the total effect (Figure 2, path Total).

*(insert Figure 1 about here)*

*(insert Figure 2 about here)*

Next, we tested whether frustration of basic needs (i.e., frustration of autonomy, competence, and relatedness) mediated the relation between workplace bullying exposure and the outcome variables. Figure 3 shows the specified structural model. Again all outcome variables were included in one model with correlated error terms. This model showed reasonable model fit (χ² = 4684.370, *df* = 1073, *p* < .001, *RMSEA* [CI_90_] = .052 [.050; .052], *SRMR* = .051, *CFI* = .899, *TLI* = .890). Workplace bullying exposure was a strong predictor for all basic need frustrations. As the inspection of the correlational analysis already suggested, there was a high multicollinearity between the different basic need frustrations (*VIF*_autonomy_ = 3.76, *VIF*_competence_ = 5.29, *VIF*_relatedness_ = 4.24). However, the variance inflation factors fell below the suggested cutoff value for extreme multivariate collinearity of *VIF* > 10 (Kline, 2016), thus allowing for estimation of the effects of all these variables. Frustration of autonomy was a significant predictor for all outcome variables except workplace deviance (Figure 4, path b1) when controlling for the other basic need frustrations competence and relatedness and the direct effect of workplace bullying exposure. Furthermore, frustration of competence was only related to work performance (Figure 4, path b1) and frustration of relatedness was a predictor for well-being and vigor (Figure 4, path b3) when controlled for other effects. These results were mirrored by the indirect effects. Frustration of autonomy significantly mediated all outcomes except workplace deviance (Figure 4, path a1*b1). Frustration of competence only mediated between workplace bullying exposure and lower work performance (Figure 4, path a2*b2). Finally, frustration of relatedness mediated the association between workplace bullying exposure and well-being as well as vigor (Figure 4, path a3*b3).

*(insert Figure 3 about here)*

*(insert Figure 4 about here)*

### Figure 1. Psychological contract violation as mediator between workplace bullying and outcomes.


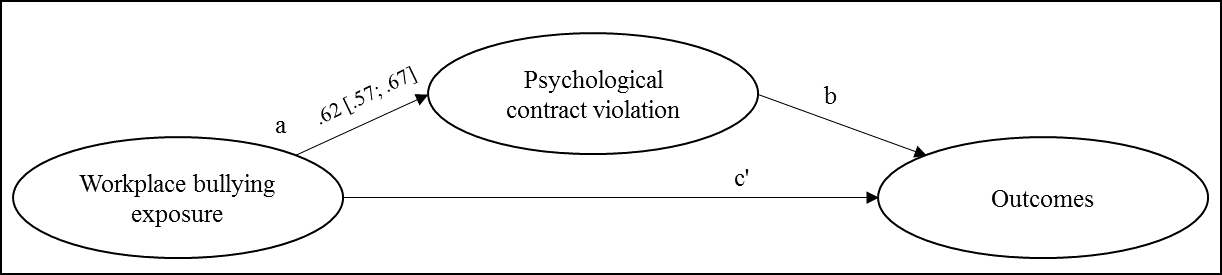


*Notes*. Standardized effects. Item-level structure of the constructs, error terms and correlations between error terms of dependent variables are not shown, for simplicity and clarity. CI_95_ based on 10,000 bootstrap samples calculated with percentile bootstrap approach; *R*²_Psychological contract violation_ = .39.

### Figure 2. Psychological contract violation as mediator between workplace bullying and outcomes: Parameters.


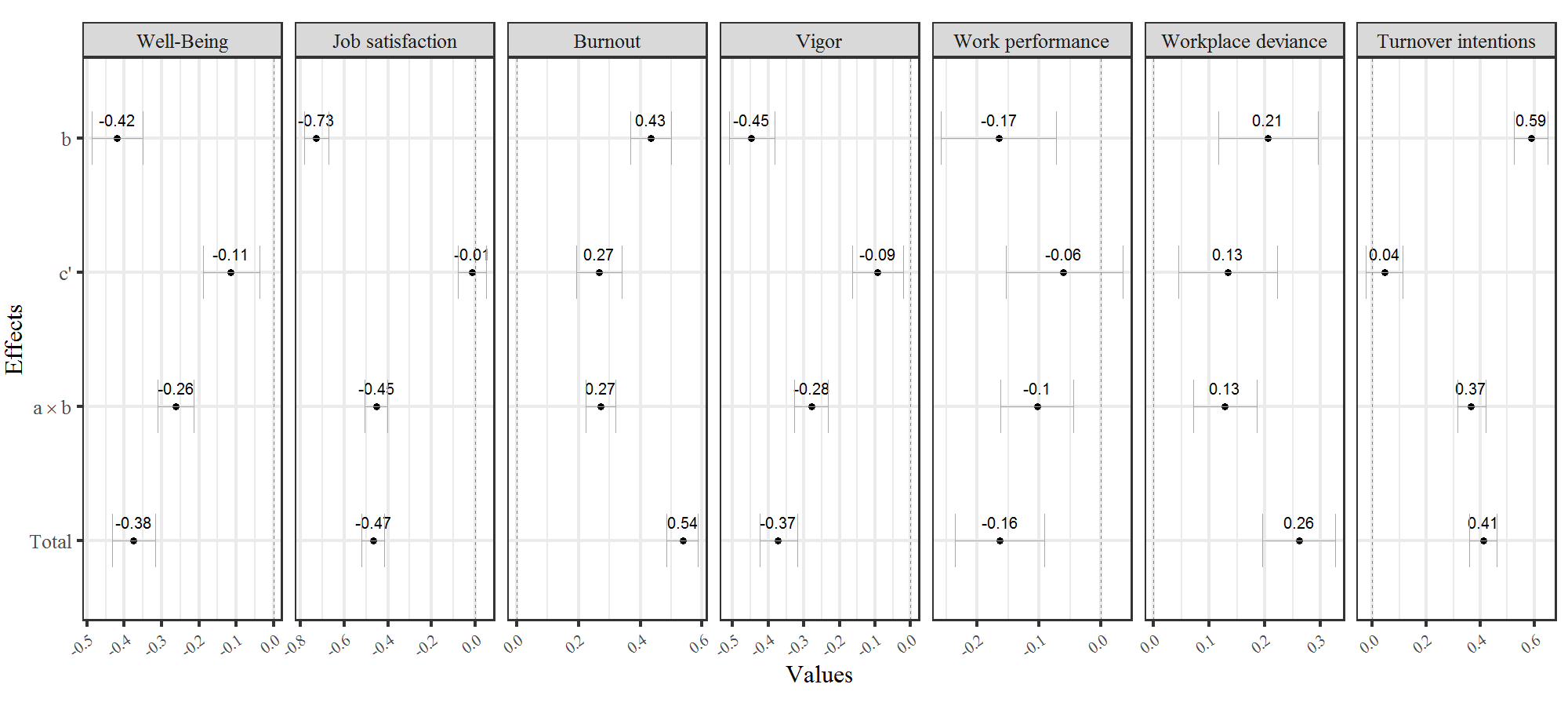


*Notes*. For paths related to “a” see Figure 2. CI_95_ based on 10,000 bootstrap samples calculated with percentile bootstrap approach; *R*²_Well-Being_ = .25; *R*²_job satisfaction_ = .54; *R*²_burnout_ = .40; *R*²_vigor_ = .26; *R*²_work performance_ = .04; *R*²_workplace deviance_ = .09; *R*²_turnover intentions_ = .38.

### Figure 3. Basic need frustrations as mediators between workplace bullying and outcomes.


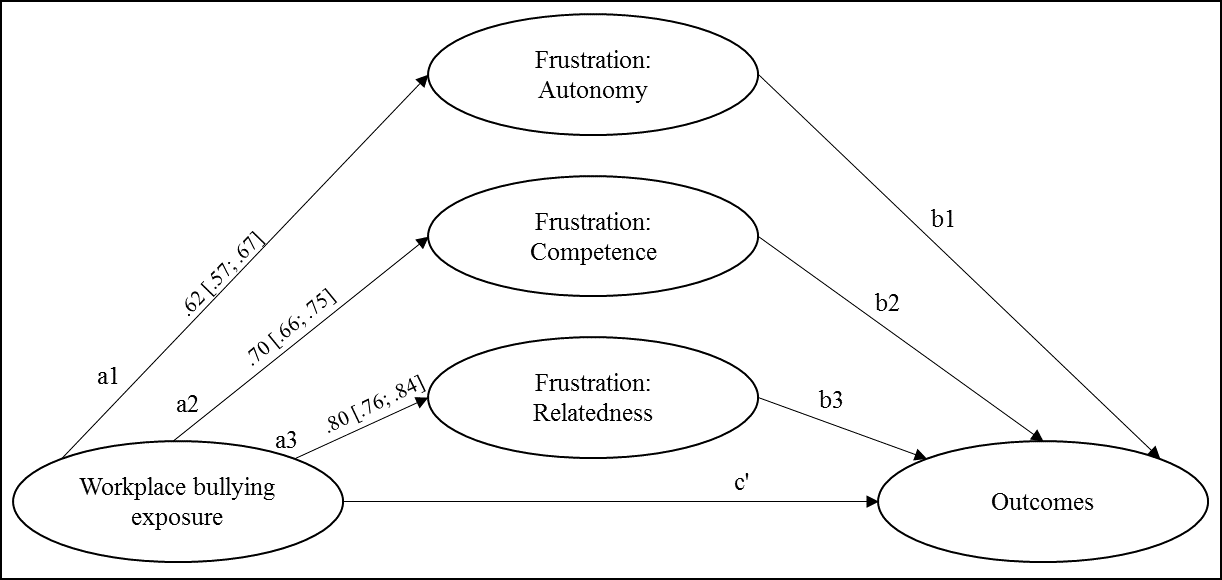


*Notes*. Standardized effects. Covariance among independent variables, item-level structure of the constructs, error terms and correlations between error terms of dependent variables are not shown, for simplicity and clarity. CI_95_ based on 10,000 bootstrap samples calculated with percentile bootstrap approach; *R*²_Frustration Autonomy_ = .39; *R*² _Frustration Competence_ = .50; *R*² _Frustration Relatedness_ = .64.

### Figure 4. Basic need frustrations as mediators between workplace bullying and outcomes: Parameters.


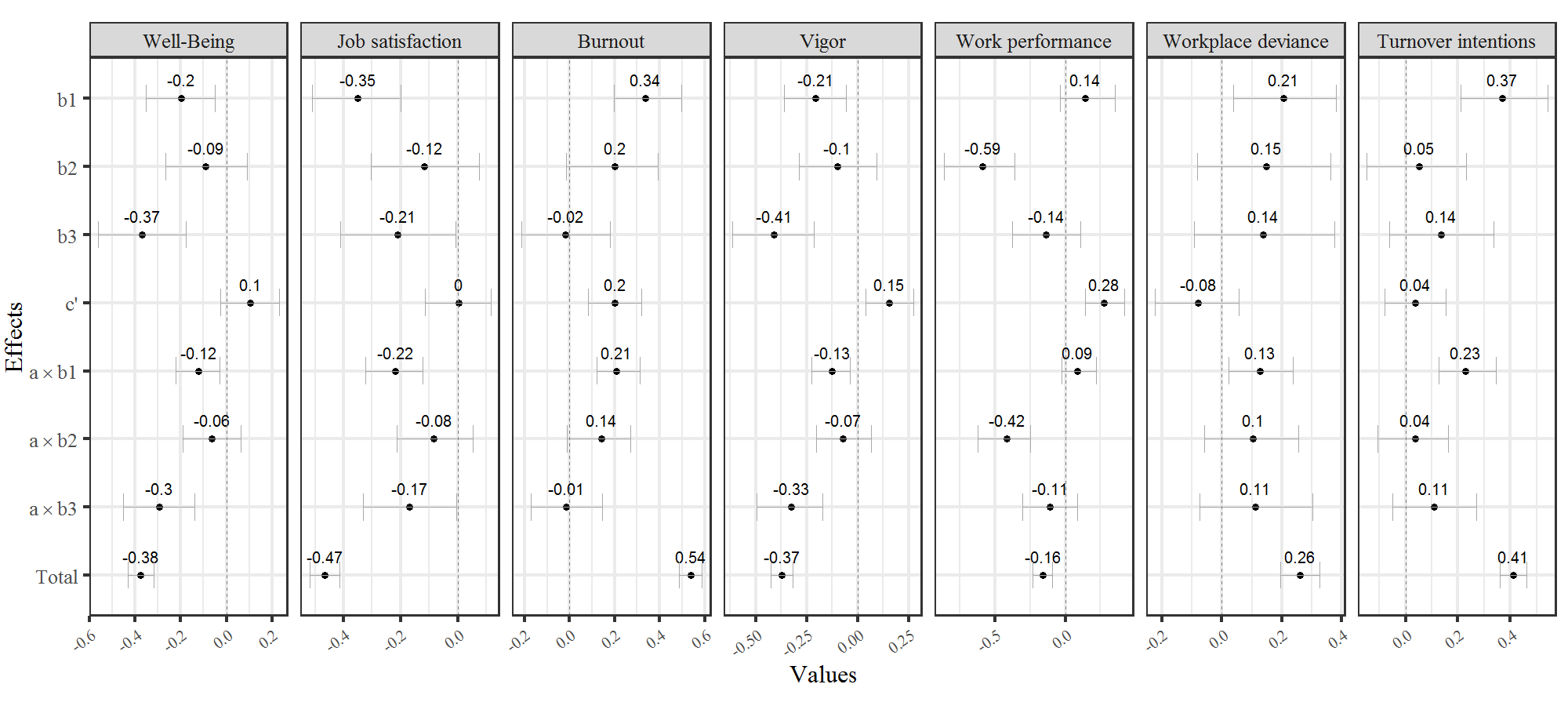


*Notes*. For paths related to “a” see Figure 4. CI_95_ based on 10,000 bootstrap samples calculated with percentile bootstrap approach; *R*²_Well-Being_ = .30; *R*²_job satisfaction_ = .41; *R*²_burnout_ = .43; *R*²_vigor_ = .32; *R*²_work performance_ = .19; *R*²_workplace deviance_ = .17; *R*²_turnover intentions_ = .31
